# African Migrant Patients’ Trust in Chinese Physicians: A Social Ecological Approach to Understanding Patient-Physician Trust

**DOI:** 10.1371/journal.pone.0123255

**Published:** 2015-05-12

**Authors:** Megan M. McLaughlin, Louis Simonson, Xia Zou, Li Ling, Joseph D. Tucker

**Affiliations:** 1 UNC Project-China and Sun Yat-sen Center for Migrant Health Policy, Sun Yat-sen University School of Public Health, Guangzhou, Guangdong Province, China; 2 Department of Geography and Planning, Sun Yat-sen University, Guangzhou, Guangdong Province, China; 3 Sun Yat-sen Center for Migrant Health Policy and Faculty of Medical Statistics and Epidemiology, Sun Yat-sen University School of Public Health, Yuexiu District, Guangzhou, Guangdong Province, China; 4 Sun Yat-sen Center for Migrant Health Policy and Faculty of Medical Statistics and Epidemiology, Sun Yat-sen University School of Public Health, Yuexiu District, Guangzhou, Guangdong Province, China; 5 UNC Project-China, Guangzhou, Guangdong Province, China; University of Washington, UNITED STATES

## Abstract

**Background:**

Patient trust in physicians is a critical determinant of health seeking behaviors, medication adherence, and health outcomes. A crisis of interpersonal trust exists in China, extending throughout multiple social spheres, including the healthcare system. At the same time, with increased migration from Africa to China in the last two decades, Chinese physicians must establish mutual trust with an increasingly diverse patient population. We undertook a qualitative study to identify factors affecting African migrants’ trust in Chinese physicians and to identify potential mechanisms for promoting trust.

**Methods / Principal Findings:**

We conducted semi-structured, in-depth interviews with 40 African migrants in Guangzhou, China. A modified version of the social ecological model was used as a theoretical framework. At the patient-physician level, interpersonal treatment, technical competence, perceived commitment and motive, and language concordance were associated with enhanced trust. At the health system level, two primary factors influenced African migrants’ trust in their physicians: the fee-for-service payment system and lack of continuity with any one physician. Patients’ social networks and the broader socio-cultural context of interactions between African migrants and Chinese locals also influenced patients’ trust of their physicians.

**Conclusions:**

These findings demonstrate the importance of factors beyond the immediate patient-physician interaction and suggest opportunities to promote trust through health system interventions.

## Introduction

Trust is an essential component of the patient-physician relationship. Patient-physician trust affects a patient’s willingness to see a physician, disclose information, and accept therapy, thus enabling the cooperation between the patient and physician that is needed for effective care [1,2]. Patients who trust their physicians are more likely to seek health care [3], adhere to therapy [3–6], and report better mental and physical health [3,7]. Trust in physicians is conditional and subject to an iterative process: patients test the trustworthiness of their physicians against their expectations [8,9]. Studies have shown that beyond physician characteristics and behaviors, characteristics of health care institutions and the health system also influence patient trust. These factors include continuity of care [10], degree of choice of physician [11], and accessibility of the physician [10,12].

Patient race or ethnicity can also have an important influence on trust. Studies in the U.S. have found that compared to white patients, African-American patients report less trust in physicians and more distrust of the health care system [6,11,13–17]. This distrust may help in part to explain racial/ethnic disparities in health outcomes by affecting treatment seeking and adherence to treatment [5,6,18]. Thus, trust is an important factor in the health system’s capacity to deliver high-quality, responsive care to minorities and marginalized groups [2].

Much of the existing literature on patient-physician trust draws on research in the U.S. and Europe [9,19]. Comparatively less has been written about trust in physicians in low- and middle-income countries, particularly among minority populations [2,20]. But health care providers in low- and middle-income countries increasingly must provide culturally appropriate services for a diverse population. South-South migration (migration between countries of the global South) accounts for a large proportion of global population movement [21]. Since the late 1990s, as Sino-African ties have deepened, bidirectional travel and migration between China and countries in Africa have increased [22]. They face a number of barriers to health care, including the cost of health services, discrimination, and a lack of interpreter services [23]. Establishing a relationship of mutual trust and providing high-quality medical care to this diverse population pose challenges for Chinese physicians. We undertook a qualitative study in Guangzhou, China in order to better understand trust relationships between African patients and Chinese physicians. Our aim was to identify interpersonal, social network, health system, and socio-cultural factors related to African migrants’ trust in Chinese physicians.

## Methods

### Setting and population

Located on China’s southeastern coast, Guangzhou is the third largest city in China with a population of more than 12 million [24]. It is part of the highly developed Pearl River Delta region of Guangdong Province, a major hub for economic development [25]. The size of the African migrant population in Guangzhou has grown dramatically since the 1997 Asian financial crisis led many African traders to leave Thailand, Indonesia, and other countries in Southeast Asia for opportunities in China [22]. Although African migrants are found in all of China’s major cities, they have historically been concentrated in Guangzhou [22,26]. More than 100,000 African migrants are estimated to live in Guangzhou alone [22,27]. Previous studies have documented an emergence of increased racist discourse in the Chinese media and online, as migration been China and Africa has increased over the last decade and a half [28].

Several surveys undertaken in Guangzhou have found that African migrants are predominately from West Africa, with Nigerians comprising the largest group [22,29]. Most Africans sampled were men who identified as businessmen and traders, who fall into two main groups: exporters who reside in Guangzhou and importers (to Africa) who make frequent visits to Guangzhou, often staying for a week to a month [22,30]. However, the population also includes a large group of students from Africa studying at universities in Guangzhou, as well as a number of female traders, businesswomen, and housewives. The socioeconomic backgrounds of African migrants span a wide range, but many are traders with limited resources who engage in small-scale, informal trade activities [30–32]. Although publicly available official figures are lacking, it is estimated that many African migrants residing in Guangzhou have expired visas [26,27], and periodic police crackdowns against undocumented African migrants in Guangzhou have been reported in the media.

Foreigners seeking care in Guangzhou have a number of health care settings to choose from: academic and public tertiary care hospitals, traditional Chinese medicine hospitals, and private clinics, including those that employ foreign physicians and cater specifically to foreigners. The latter tends to be the most expensive, while academic and public hospitals tend to be the more affordable option. In a typical Chinese hospital, a patient is assigned to a doctor at the time they register for care, on the same day as their visit. Rather than scheduling an appointment with in advance, patients choose an appointment slot with one of the physicians on duty that day. They are generally required to pay a consultation fee at the time of registration. If diagnostic tests or procedures are needed, the patient is required to pay for these procedures before receiving them. Because they lack health insurance, African migrants make these payments out of pocket. In China, foreign migrants generally do not have access to insurance. Health insurance schemes offering coverage for foreigners have been piloted in Beijing, but have not yet been widely implemented [33]. Although the initial consultation fee at public hospitals is often a small amount of money, the cost of laboratory tests, procedures, and medications can be prohibitively expensive, particularly for small-scale traders. Africans living in China face a variety of barriers to accessing existing health care services in Chinese health facilities. Interpersonal discrimination, different expectations for medical care, tenuous legal status, and communication problems with Chinese health care professionals who do not speak foreign languages act as barriers to Africans’ utilization of health care services in China [23]. The absence of formal interpreter services in Chinese health facilities creates further challenges for Africans accessing care in China [23].

### Design and sample

We employed a qualitative approach using semi-structured, in-depth interviews with 40 African migrants. We chose qualitative methods because we sought to generate a detailed, context-specific understanding of African migrants’ trust in Chinese physicians [34]. We continued to conduct interviews until we reached theoretical saturation. We used purposive sampling for the recruitment of African migrants to ensure that the sample included 1) a diversity of countries in West Africa, East Africa, and Southern Africa, 2) men and women, and 3) long-term residents of Guangzhou (more than one year) and short term residents or visitors (one year or less). Prior to beginning recruitment we decided that it was important to recruit participants who varied according to region of origin, sex, and time in Guangzhou. Initially we recruited participants at random, resulting in a sample that was heavily West African and male. As our recruitment continued, we preferentially sought out women and participants from countries outside West Africa.

Prior to participant recruitment, we conducted formative research to better understand the African population in Guangzhou and potential recruitment sites. We met with ten key informants, including African community leaders, African students, African traders, imams at the local mosques, and an African physician practicing in Guangzhou. We toured potential recruitment sites with key informants and conducted three weeks of observation at these sites. Key informants were not included in the sample of 40 participants interviewed, and information they provided was not included in the qualitative analysis.

African migrant participants were subsequently recruited from three types of locations: 1) trading markets, 2) religious institutions, and 3) universities. Previous research revealed that African traders were concentrated primarily in two locations—in the Xiaobei area of Yuexiu district and the Sanyuanli area of Baiyun district [22,26,27,29,30]. These areas, located near the Guangzhou Railway Station, are convenient for transport and provide cheap accommodation [30]. When recruiting traders, we took into consideration the daily fluctuations in trade activity and recruited on weekday afternoons, when the markets are most active.

We introduced the project to six religious organizations in Guangzhou. With the permission of the local religious leaders, we approached potential participants immediately before and after the main weekly services at four of these organizations. We recruited participants from two officially recognized mosques in Guangzhou that serve the largest number of Africans, as reported by key informants. We also selected two churches: a large Catholic church that holds an English-language Sunday service and a non-denominational Christian church with membership limited to foreigners. Additionally, African students were recruited through our existing foreign student contacts at a highly ranked national public university, a regional university of technology, and a regional medical school. Inclusion criteria for participation were proficiency in English, age 18 years or older, citizenship in a country in Africa, and experience receiving health care in China. We excluded 15 participants, with lack of English proficiency being the primary reason for exclusion. Those who were primary French or Portuguese speakers who were able to speak English proficiently were included in the study. We identified more than 40 potential participants who met the inclusion criteria. Some eligible participants, however, declined to participate in the study. The primary reason given for refusal was being too busy to participate; other reasons included not wanting to take the time to do the interview, believing their English speaking skills were insufficient, or feeling uncomfortable being interviewed.

### Data collection

The data collection was completed between January and May 2013. The semi-structured interview guide consisted of open-ended questions to foster discussion of experiences receiving health care in China. During the initial interviews, issues related to patient-physician trust emerged. We incorporated these topics into a revised version of the interview guide that was used in subsequent interviews and form the basis of this analysis. African migrants were asked to narrate their most recent health care encounter in China, and they were asked to describe how they decided whether or not they could trust the physician.

The first author led the interviews, while a research assistant recorded notes. Participants were offered a free meal for participation in the study. Each interview lasted approximately 30–45 minutes. Interviews were audiotaped if participants provided consent and subsequently transcribed; otherwise, notes recorded during the interviews were used for the analysis.

### Ethics statement

Study procedures were approved by the Institutional Review Board at the University of North Carolina Chapel Hill and the Guangdong Provincial Dermatology Hospital. Participants provided written informed consent to participate in the study.

### Theory

Patient-physician trust includes both interpersonal trust—trust in an individual physician—and impersonal trust—trust in physicians in general [1,2]. These types of trust are inter-related. Impersonal or institutional trust enables a patient to trust a new physician, and trust relations built through interpersonal interactions with physicians help to sustain impersonal trust in physicians in general and the institutions they represent [1,35–37]. We focused on interpersonal patient-physician trust because the importance of this type of trust for patient care outcomes has been demonstrated [4–7]. Drawing from Hall et al. [19] and Russell [37], we define trust as the optimistic acceptance of a vulnerable situation in which the truster believes the trustee will care for the truster’s interests and has the competency to do so.

Given the inter-related nature of interpersonal, impersonal, and institutional trust in the health care system, we sought to identify not only factors at the patient-physician level that may influence African patients’ trust in Chinese physicians, but also structural and socio-cultural factors. As Wuthnow argues, “trust does not depend only on judgments one person makes about another, but also on assumptions that emerge from the context in which relationships take place, on expectations derived from previous relationships, and on criteria for making judgments that are deemed legitimate by the actors involved” [38].

**Fig 1:**
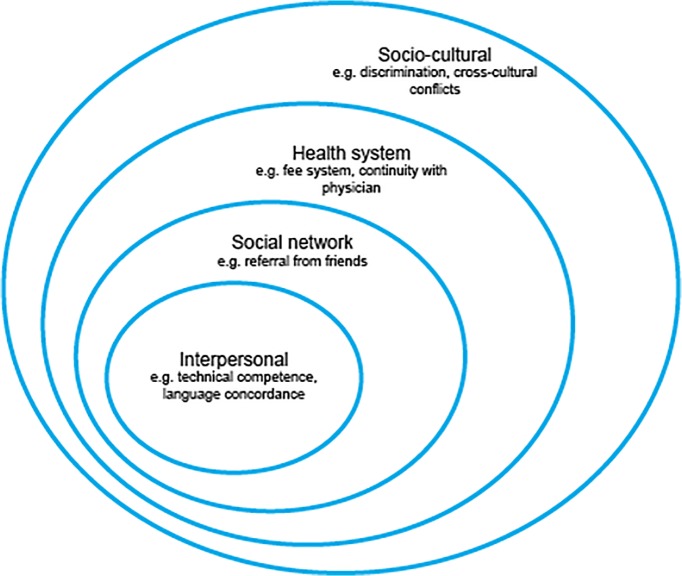
Social ecological framework for African migrant patients’ trust of Chinese physicians.

We used a modified version of the social ecological model [39,40] as a framework for organizing the themes that emerged from the interviews (Fig 1). The social ecological model has been widely used in the field of public health to analyze factors affecting health behavior and health promotion interventions. This model recognizes that behavior is affected by not only individual factors but also multiple spheres of influence in an individual’s social environment. In the modified social ecological model we used for this analysis, we included the following levels: interpersonal, social network, health system, and socio-cultural.

### Analysis

Two researchers reviewed the transcripts and inductively developed a preliminary list of codes representing the main themes. These codes were categorized into one of four levels of a modified social ecological framework. Using the initial code structure, three individuals independently coded transcripts and cross-checked coding until a consensus was reached about the code structure. The data were analyzed using Atlas.ti version 7 (Scientific Software Development GmbH, Berlin, Germany).

## Results

Among the 40 African migrant participants, 29 were traders, 9 were students, one was a restaurant worker, and one was a housewife (supporting information, S1 Table. Demographic characteristics of African migrants interviewed). Fourteen African countries were represented. Nigerians constituted the largest number of participants (16), followed by participants from Guinea (5), Zimbabwe (3), and Ghana (3). The average age was 34 years, and 90% of participants were male, reflecting the greater proportion of men in the African migrant population. Most had limited or no Chinese language proficiency. The participants had a wide range of experience with the Chinese health care system, ranging from one health care encounter to years of health care visits. Participants primarily reported using modern medicine; a small number also reported using traditional Chinese medicine. Although most of the African migrants in our sample described challenges in their interactions with Chinese physicians, a subset reported high levels of trust in their physicians. This trust was influenced by a complex set of factors at the interpersonal, social network, health system, and socio-cultural levels. Key supporting quotes for these themes are provided in S2 Table (supporting information, S2 Table. Quotes supporting study themes).

### Interpersonal level

Factors at the patient-physician level were the most frequently discussed factors influencing participants’ trust of physicians. Often participants entered the patient-physician interaction with ideas about the trustworthiness of Chinese physicians in general based on the experiences of others in their social networks, encounters with Chinese citizens outside the health care setting, and previous experiences with Chinese institutions. However, participants evaluated and tested the physician’s trustworthiness during the clinical interaction. Two participants stated that they had no choice but to trust the physician they sought care from, but most participants reported critically evaluating the trustworthiness of the physicians they encountered. Many reported trust in the individual physicians they saw, even as they described mistrust in Chinese physicians in general or the broader health system.

Among the most frequently cited factors at the patient-physician level was the physician’s interpersonal treatment of the patient. When discussing physicians they trusted, participants used descriptors like “caring,” “kind,” and “looks after you.” They trusted physicians who demonstrated willingness to answer questions and who provided encouragement to them. Several participants described their relationship with these physicians as one of friendship. In contrast, other participants described physicians who failed to demonstrate positive interpersonal treatment of patients, by shouting, ordering them around, or ignoring them. One student described a colleague whose trust in the physicians and the medication they prescribed was undermined when his doctor ignored him and people at the clinic laughed at his Chinese language skills. On his way out of the hospital, he discarded the medication he had been prescribed.

In evaluating the trustworthiness of their physicians, participants also considered whether the physicians were qualified. Participants used different strategies to assess the technical competence of their physicians, but often it was based on the outcome of care. One participant reported trusting his doctor in China because after failing to receive a diagnosis in his home country, the doctor in China was able to diagnose him. Others judged their physician’s technical competence by the questions asked during the history and physical exam. For others, their trust was affected by how well their physician was able to answer their questions. Several participants worried specifically that the physician might not be prepared to treat diseases that are more common in Africa, particularly malaria. One trader from Nigeria explained that he evaluated his physician by asking about his time spent abroad.

Participants also reported that their perceptions of their physician’s commitment affected their trust. They used descriptors like “committed to their work,” “dedicated to their job,” “love their job,” and “trying their best” to describe physicians they trusted. In contrast, several participants explained that their physicians seemed to care more about making money than their well-being.

Finally, patient-physician language concordance played an important role in patient trust. Nearly all participants reported substantial language barriers during their health care visits. Participants sometimes brought Chinese friends, business contacts, or significant others to interpret. None encountered professional interpreters in the hospitals or clinics they visited. Some explicitly stated that they did not trust doctors who were unable to speak English, or conversely that they trusted those who could speak English well. One woman, who interacted with several different doctors during a routine check up, explained that she did not trust those who could not speak English because she had no way to assess the care they provided. Thus, language concordance was an important foundation on which trust was built. How physicians responded to language discordance also affected trust. Some participants with limited Chinese proficiency explained that they encountered physicians who did not want to take the time to try to understand them. Participants were more likely to trust those physicians who demonstrated an effort to make sure that they understood, such as drawing pictures to explain concepts.

### Social network level

Participants’ social networks in Guangzhou had both positive and negative effects on their trust in physicians. Many reported difficulty navigating the health system in China, beginning with finding a hospital or clinic and choosing an appropriate doctor. Where possible, participants usually drew on their various networks, often relying on friends from their home countries, whom they referred to as brothers and sisters. Most of the countries represented in our sample had a local union or community organization that connected traders, students, and other migrants to their compatriots. Traders also sought recommendations from Chinese business colleagues and Chinese wives or girlfriends. Students also relied on fellow students or resources provided by the foreign student office at their university. Several participants reported that they were more likely to trust their physician if he or she had been recommended by a friend or colleague. In turn, participants who found a physician they trusted often reported referring that physician to others.

On the other hand, participants who reported an underlying mistrust of Chinese physicians were often influenced by negative experiences of their African friends. When discussing physicians whom they did not trust, participants not only drew on their personal experiences but also frequently recounted negative experiences of their friends. One student who was visibly suffering from an eye infection explained that he preferred to wait until he returned home to have his eye examined. Despite having had a positive encounter with several Chinese physicians during a check-up required for his student visa, his friends’ and classmates’ experiences being misdiagnosed or ill-treated had undermined his trust in Chinese physicians.

### Health system level

At the health system level, two primary factors influenced African migrants’ trust in their physicians: the payment system and lack of continuity with any one physician. With the exception of students, most participants reported that they did not have health insurance in China and were forced to pay out of pocket. The majority reported having to pay for care at the time of registration, before seeing the doctor, in contrast to practices in their home countries. For some, this policy led them to question the priorities of their physicians, particularly when this practice differed from payment practices in their home countries. Other participants were more concerned about having to pay separately at each step of the process—for the consultation, diagnostic tests, intravenous drips, and medications—which seemed to emphasize money throughout the encounter. Awareness about the fee-for-service nature of the payment system led some participants to question the motivations of their doctors. A notable example was when they were prescribed what seemed to be an unnecessarily large number of medications. Some believed that the fee-for-service system motivated providers to act against the patient’s interest by encouraging unnecessary prescriptions.

Participants also described an inability to make appointments with doctors in China, which prevented continuity of care with any one physician. Instead, those who had returned to the same health facility several times generally reported seeing a different doctor each time. For some, this meant that they were unable to develop a relationship of trust with a physician over time. Those participants who reported that the lack of physician continuity affected their trust in the physicians contrasted this lack of continuity to their experiences in their countries of origin. One participant explained that he trusted his doctor at home because he grew up receiving care from him. He then explained that there was one doctor in China whom he had seen more than once, and he felt that the care he received improved the more he saw that doctor.

Responding to this gap in the system, some participants proactively sought to establish a long-term relationship with a physician with whom they had a positive initial experience. Often this meant learning the physician’s work schedule and only seeking care on days when they were on duty. Since there was no way to formally schedule appointments through the hospital administration, at the end of each visit one participant informally scheduled each subsequent visit directly with her obstetrician. Four participants said they requested the physician’s phone number so that they could call or text ahead of time to make sure the physician would be available or to request informal referrals to other types of specialists if needed.

### Socio-cultural level

The broader socio-cultural context in which Africans and Chinese interacted also shaped participants’ trust in Chinese physicians. Many participants described feeling marginalized, mistreated, or singled out because of their race, both inside and outside health care settings. They recounted stories of physicians who did not want to accept them as patients, who avoided touching them, and who gave preferential treatment to Chinese patients. One female trader who had lived in China since the mid-1990s described an improvement over the last two decades in how she was treated by Chinese physicians. Nevertheless, her story of a physician in Shanghai in the mid-1990s who refused to perform a physical exam on her was echoed in recent migrants’ stories of present-day Guangzhou. These individual instances of perceived discrimination in the patient-physician encounter were often interwoven in the participants’ narratives with discrimination they faced in other public places. Prior experiences with discrimination outside health care settings in China led a subset of participants to interpret certain physician behaviors, such as failing to do a physical exam or refusing to accept a patient, as racially motivated.

Beyond experiences of discrimination, conflicts with Chinese during business transactions or daily life undermined trust in physicians, particularly among traders. Some participants explained their lack of trust in their physicians by referring to incidents when they had been cheated or lied to during business transactions. One participant described refusing to let a Chinese physician operate on his eye for fear that they would make a mistake and not admit to it. He elaborated explaining his frustration that people in China do not own up to their mistakes. As evidence of this, he recounted a story of a taxi driver who mistreated him and lied to the police about the encounter. Perceptions of Chinese-manufactured products being of poor quality were also mentioned as evidence for why Chinese health care could not be trusted.

## Discussion

This study identifies factors affecting African migrants’ trust in physicians in China. Our findings build on the growing global literature on the role of trust in patient-physician relationships, which is essential for informing quality improvements in health service delivery. We found that trust was influenced by a complex set of factors that included not only physician behavior during the clinical encounter but also aspects of the Chinese health system and the socio-cultural environment.

Similar to previous studies [8,9,41–43], we found that patients evaluate and test their physicians’ trustworthiness during the clinical interaction. The factors we identified at the patient-physician level—interpersonal treatment, technical competence, and perceived commitment—are similar to those that have been reported in the U.S. and Europe and among Chinese patients [8,12,42–44]. In addition, we found that language concordance was a key factor at the interpersonal level. In cases where the physician lacked proficiency in English, patients had difficulty evaluating the trustworthiness of their physician based on other factors at the interpersonal level.

For some participants, a focus on payment early and throughout the encounter created mistrust. Studies in low- and middle-income countries have found that fee systems have an important influence on patient trust by affecting patients’ perceptions of health care providers’ motivations [20,37,45,46]. As Gilson [20] explains, patients sometimes view financing mechanisms as signals of value within the health system. The type of fee system can affect patients’ perception of whether profit seeking or caring is prioritized. The fact that payment was required before the consultation with the physician and that patients were charged repeatedly throughout the visit led them to believe that the physician’s first priority was earning money, not caring for the patient.

Lack of continuity of care in Chinese health facilities was an important factor affecting trust. Several previous studies in the U.S. have found that continuity with a single physician is associated with greater trust in physicians [10,11,47]. Several participants in our study proactively sought to establish a long-term relationship with one physician by requesting the physician’s phone number and learning his or her work schedule. These repeated visits provided an opportunity for building interpersonal trust over time. A similar desire for continuity among patients has been reported by studies in Thailand [45] and Sri Lanka [37], where similar to Chinese public hospitals, public facilities cannot guarantee patients will see the same doctor over time.

Experiences of racial discrimination also affected African migrants’ trust in Chinese physicians. Studies among African-American patients in the U.S. have demonstrated the important relationship between prior experiences of discrimination and distrust of physicians [17,48,49]. We found that discrimination inside and outside health care settings were interwoven in participants’ narratives about their health care experiences in China. Although some of the physician behavior that was interpreted as racially motivated, such as demonstrating impatience or failing to do a physical exam, may have been the result of language barriers or workload pressures, but what was important for patient trust was the perception of discrimination. The experience described by African migrants in our study has commonalities with the experience of China’s internal migrant population, which also faces limited access to health care and discrimination leading to distrust [50,51].

Our findings about factors influencing African migrants’ trust in Chinese physicians have implications for health policy reform in China. In particular, the relationship between patient trust and factors at the health system level suggests strategies for promoting trust and improving the quality of care for African migrants through health care delivery interventions. First, given that language concordance was widely identified as a basic foundation for building trust, the availability of professional interpreters in person or by phone is critical. Our findings also suggest that reforming the fee structure so that patients make a single payment after receiving care could improve trust by removing the focus on payment during the clinical encounter. Pilot programs allowing patients to receive care before payment have been implemented in more than 20 Chinese hospitals [52]. A hospital in Shenzhen has also piloted a care delivery model in which patients pay a single upfront fee that covers the consultation, diagnostic tests, and prescriptions received during the visit [53]. Studies in some of these settings have found that the reformed payment policy has improved the relationships between Chinese patients and physicians [54–56]. Lastly, implementing a primary care model that allows patients the opportunity to develop a long-term relationship with a physician could be important. This model is already being piloted at sites in Shenzhen, Beijing, and Shanghai [53,57,58]. Expanding this model to Guangzhou in conjunction with interpreter services and outreach to African community leaders might help to promote trust in physicians among African migrants.

The African migrant population in Guangzhou is diverse, and it is difficult to generate findings that are generalizable for this population. We sought to capture this diversity in our study through purposive sampling. Due to the sensitivity of the topic, we did not specifically ask participants about their visa status, and discussion of visas and immigration concerns arose infrequently in our interviews. Two participants, however, mentioned that they did not have valid visas. Migrants without current documentation may have been less likely to participate in our study, a challenge that other researchers studying the African population in Guangzhou have reported [22]. Additionally, we did not recruit African migrants from Francophone or Lusophone countries who were unable to speak English. These are likely to be some of the most marginalized migrants, and research on their health care experiences in China is needed.

As China pursues ongoing national health reform [59], the experience of African migrant patients needs to be considered when designing interventions to improve the quality of patient-physician relationships. Additional research and pilot studies are needed to better understand the potential for measures such as professional interpreter services, payment reform, and primary care models to improve African migrants’ trust in Chinese physicians.

## Supporting Information

S1 Table Demographic characteristics of African migrants interviewed. (PDF)

S2 Table Quotes supporting study themes. (PDF)
