# Supplementary material for: African Migrant Patients’ Trust in Chinese Physicians: A Social Ecological Approach to Understanding Patient-Physician Trust
Source: PLoS One. 2015 May 12;10(5):e0123255. doi: 10.1371/journal.pone.0123255 (PMC4428824; doi:10.1371/journal.pone.0123255)
Supplement: S1 Table — (PDF) [file pone.0123255.s001.pdf]

Table S1. Demographic characteristics of African migrants interviewed

No.	Country of origin	Time in China	Sex	Age (years)	Occupation	Education level	Mandarin proficiency	English proficiency	Other languages spoken
1	Nigeria	1 week	M	40	trader	bachelor's	none	convesational	Yoruba
2	Guinea	5 years	M	21	trader	high school	advanced	intermediate	Fula, French
3	Mali	6 months	M	29	trader	high school	basic	intermediate	Bambara, French
4	Mali	has been visiting China for several years, usually making a visit every 2 months	M	44	trader	high school	none	intermediate	Bambara, French
5	Guinea	5 years	M	35	trader	primary school	none	intermediate	Susu, French, Yoruba
6	Guinea	has been visiting China for 13 years, usually making trips twice a year	M	42	trader	high school	very basic	intermediate	Susu, French, Malinké
7	Guinea	has been visiting China for 8 years, longest stay was 3 years	M	52	trader	elementary school	none	intermediate	French
8	Niger	4 years	M	32	trader	some university	intermediate	intermediate	French
9	Sierra Leone	17 years	F	41	trader	master's	fluent	fluent	
10	Guinea	7 years	M	36	trader	bachelor's	basic	conversational	Malinké, French, some Arabic
11	Senegal	9 years	M	32	trader	some university	very basic	intermediate	Wolof, French
12	Nigeria	2 years, 6 months	M	36	trader	some high school	very basic	intermediate	Igbo
13	Nigeria	12 years	M	36	trader	some university	intermediate	conversational	Igbo, French
14	Nigeria	5 years	M	40	trader	high school	basic	intermediate	Igbo
15	Nigeria	6 years	M	35	trader	some high school	basic	intermediate	Igbo
16	Nigeria	5 years	M	36	trader	high school	basic	intermediate	Igbo
17	Nigeria	1 year	M	27	trader	some university	basic	intermediate	Igbo
18	Nigeria	has been visiting for 7-8 years,	M	40	trader	some university	very basic	conversational	Igbo

19	Nigeria	makes 3-5 month-long trips each year has been visiting China 2-3 times per year for last 2 years	M	29	trader	high school	very basic	intermediate	Igbo
20	Nigeria	6 years	M	33	trader	bachelor's	basic	conversational	Igbo
21	Nigeria	less than 1 year	M	29	trader	bachelor's	advanced	intermediate	Igbo, Hausa
22	Nigeria	11 years	M	43	trader	high school	basic	conversational	Igbo, some French
23	Ghana	11 years	M	39	trader	bachelor's	advanced	fluent	Twi, three other Ghanaian languages, some Turkish and Russian
24	Nigeria	9 years	M	38	trader	bachelor's	advanced	fluent	Yoruba
25	Nigeria	has been visiting China for 6 years, has lived in China continuously for 2 years	M	31	trader	primary school	basic	intermediate	Igbo
26	Ethiopia	1 year, 2 months	M	23	trader	master's	none	conversational	Amharic, Oromigna
27	Kenya	2 years	M	53	trader	some university	basic	conversational	Swahili, some Arabic, some German
28	Nigeria	5 years	M	33	trader	high school	basic	intermediate	Igbo
29	Nigeria	1 year	M	30	trader	high school	very basic	intermediate	Igbo
30	Niger	1 month	M	37	trader/student	bachelor's	basic	intermediate	French, some Hausa, some Ghanaian languages
31	Mozambique	6 years	M	30	trader/student	master's	intermediate	fluent	Cisena
32	Zimbabwe	4 months	M	23	student	bachelor's	intermediate	fluent	Shona
33	Zimbabwe	9 months	F	28	student	bachelor's	very basic	fluent	Shona
34	Zimbabwe	9 months	M	36	student	bachelor's	very basic	fluent	Shona
35	Ghana	1 year, 10 months	M	27	student	bachelor's	none	fluent	Twi
36	Democratic Republic of Congo	6 years	M	25	student	bachelor's	advanced	conversational	French, Lingala, Swahili, a little Russian and

									Spanish
37	Gambia	1 month	M	28	student	high school	none	intermediate	Mandinka, Wolof
38	Gambia	less than 1 month	M	25	student	vocational school	basic	intermediate	Mandinka, Wolof
39	Uganda	2 years	F	28	restaurant worker	bachelor's	none	intermediate	Tooro, Swahili, some French
40	Ghana	10 years	F	33	homemaker	bachelor's	basic	fluent	Twi
