# Supplementary material for: African Migrant Patients’ Trust in Chinese Physicians: A Social Ecological Approach to Understanding Patient-Physician Trust
Source: PLoS One. 2015 May 12;10(5):e0123255. doi: 10.1371/journal.pone.0123255 (PMC4428824; doi:10.1371/journal.pone.0123255)
Supplement: S2 Table — (PDF) [file pone.0123255.s002.pdf]

Table S2. Quotes supporting study themes

Level	Theme	Quote
Patient-physician	Interpersonal treatment	"And so aside being a patient, she [the physician] also, you know, takes you in as a friend. And she gives you the best advice she can, you know? She's always trying to encourage you. You know I told her I was scared when the baby was big, she always told me no, no, no, you can do it, you know? I know you can do it, even if the baby is big, you don't need to be scared, you know? So I trusted her. I knew whatever [treatment] that she gave me [would] do me good."<br><br>"He [my friend] thought that the doctor didn't give [him] much attention. So how could he trust the medication? Maybe the doctor gave him medicine just to make him leave."
	Technical competence	"But I believe he [the physician] was qualified. That's why I felt comfortable. The questions he was asking and the referrals he was trying to make. So I kind of trusted what he was telling me… Just how I feel in my body, my background, and my age, and all that. Because for a medical doctor who doesn't ask your age and your weight, he's heading to nowhere. He has to know at least a little bit of you… So if a doctor does not ask you those questions, I rule him out completely."<br><br>"Ask whether he [the physician] has traveled outside. If you are dealing with treating foreigners, you must have traveled outside. Or you must have read something about the foreigners. Because there are different kinds of diseases. In Africa, we have different diseases. If you don't know about African diseases, then how would you treat me?"
	Perceived commitment	"For me, especially this Dr. [X], I trust her so much, because I think she's not doing the work for fun. I think she's doing it with passion. I think this is really what she wants to do."
	Language concordance	"I can trust some [physicians], but not everyone. Because if you cannot understand English, what will you tell me? ... I'll just say that I'll trust the person that knows how to speak English very well. A person who can say that you don't have sugar, a person who can say that your heartbeat is very fast. Because she can really explain what she has examined, or what she has tested from my body."<br><br>"I don't think I can see any doctor better than her. She's very good at what she does. And when you meet the doctor, she'll explain everything to let you understand, even if she has to draw. She would draw [pictures] to you to explain."
Social network	Referrals from friends or colleagues	"If I came to you, you treat me good, you are the winner, for everybody. I will tell all my brothers, hey I know this lady, she's a very good lady. No one will go [to] any other place."
Health system	Fee-for-service system	"And in China, for the medicine, for the hospital, everything is [about] the money. If you don't have money, you cannot go [see the doctor]. Always the money… You know, in my country, if the people are sick, checking him is no problem… But in China, you cannot look, you must pay the money first. They don't mind [if] you are really, really sick, you must first go to pay this money. And after [the] doctor comes to see you."
	Lack of physician	"Also, back home this doctor was treating me since I was maybe too young until I grow up, so I trust him… In China it's different. You just

	continuity	go to the doctor, you don't know him."<br><br>"China does not have a good referral system. If you are sick, you have to randomly go to a hospital. If you are lucky to find a good doctor, but you may not be able to find. And then this time if you find out that you didn't have this type of problem after all, you have to look for another."
Socio-cultural context	Racial discrimination	"It was worse before when they [Chinese physicians] didn't know who we were. Because we were like monsters, we were not known here. Africans, when first came. And it was hard for them to touch us, our skin, because they're scared that maybe our skin is diseased or have some problems… But since they start getting to know who we are, things started changing. Yeah, and when we go to the hospital now, they take care of you."
	Cross-cultural conflicts	One participant described refusing to let a Chinese physician operate on his eye "because they will do mistakes, and they will claim they don't know what they are doing, and maybe they will cut me blind. And they won't even show you their records of this." He elaborated, "Because they're China. You know, it's not making the mistakes that's my problem, but accepting the mistakes they do."<br><br>"China is known to be having a lot of fake items. So even the doctor himself could be fake, you understand? So it starts from there. You have to think these people are, everybody says they are trying to fake things, they copy things, even the doctor could be also an impostor, the doctor. You don't just trust them a hundred percent first, until you listen to most of them."
